# P01-13 A pilot study benchmarking physical activity policy actions in Europe: Lessons learned from the CO-CREATE project

**DOI:** 10.1093/eurpub/ckac095.013

**Published:** 2022-08-29

**Authors:** Margarita Kokkorou, Kate Oldridge-Turner, Ioana Vlad, Diva Fanian, Arnfinn Helleve, Anne-Siri Fismen, Sonia Malczyk, Janetta Harbron, Knut-Inge Klepp, Kate Allen

**Affiliations:** 1 World Cancer Research Fund International, London, United Kingdom; 2 Norwegian Institute for Public Health, Oslo, Norway; 3 Division of Human Nutrition, University of Cape Town, Cape Town, South Africa

**Keywords:** benchmarking tool, policy framework, policy design, physical activity, policy index

## Abstract

**Background:**

Global research shows a strong link between physical activity and risk of developing non-communicable diseases. To increase physical activity levels, governments must design and implement a comprehensive set of policy actions across a range of areas. To aid governments in this process, the MOVING benchmarking tool was developed to assess the design and strength of countries' policies in promoting physical activity, as well as gaps in policy action.

**Methods:**

The MOVING benchmarking tool was developed using a consultative process that reviewed evidence on physical activity policy design, existing benchmarking tools and built on the policy areas of the MOVING framework. The tool values the strength of policy design based on policy attributes that are evidence-based and aspirational - regardless of whether any countries have implemented them. The tool was applied to a set of physical activity policies from five countries participating in? CO-CREATE?, a European Commission-funded project: Netherlands, Norway, Poland, Portugal and UK. Physical activity policies that are currently implemented in these countries were identified based on a comprehensive country scan, with a set methodology. A set of policies from each country corresponding to benchmarks on access to quality public open space and green spaces and community and mass participation initiatives were analysed. The policies were assessed by applying the benchmark corresponding to the relevant policy area

**Results:**

The benchmarking tools easily identified the strengths and weaknesses in the design of each policy. Thus, the benchmarking tool identified where there was scope for improvement in specific policy area for each country, such as walking and cycling infrastructure and active transport. It also allowed comparisons between countries for specific policy areas. Further, by allowing a fast assessment of many physical activity policies, the benchmarking tool enabled an analysis of the interplay of single policies and draw conclusions about the overall policy environment in the selected countries.

**Conclusion:**

The MOVING benchmarking tool can inform the development and implementation of policies which promote physical activity. It can be used by government policy-makers, researchers and civil society organisations to identify areas of physical activity policy that require government action. The scores generated by the benchmarking tool will be amalgamated into an overall policy index for 27 European countries.

